# Supplementary figures and images for: Sex Differences in Expression of Pro-Inflammatory Markers and miRNAs in a Mouse Model of CVB3 Myocarditis
Source: Int J Mol Sci. 2024 Sep 6;25(17):9666. doi: 10.3390/ijms25179666 (PMC11395254; doi:10.3390/ijms25179666)

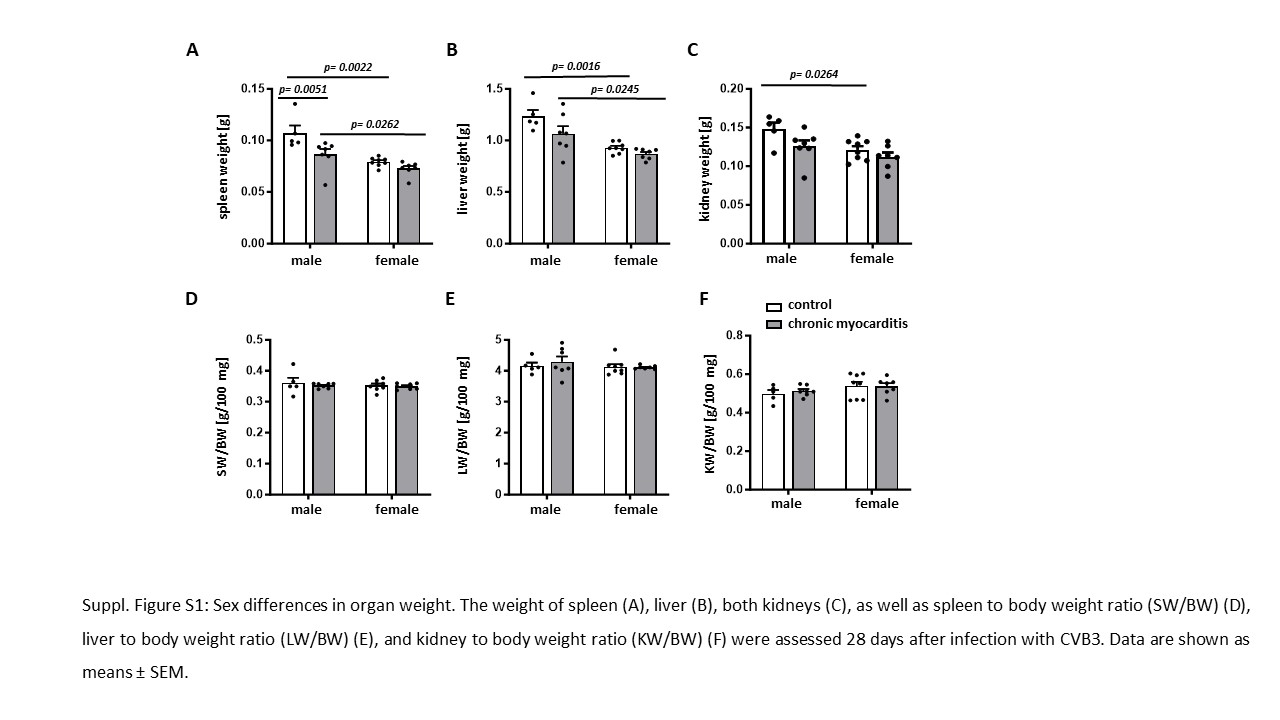

Supplement: Supplementary file 1 [file ijms-25-09666-s001.zip › Figure S1.JPG]

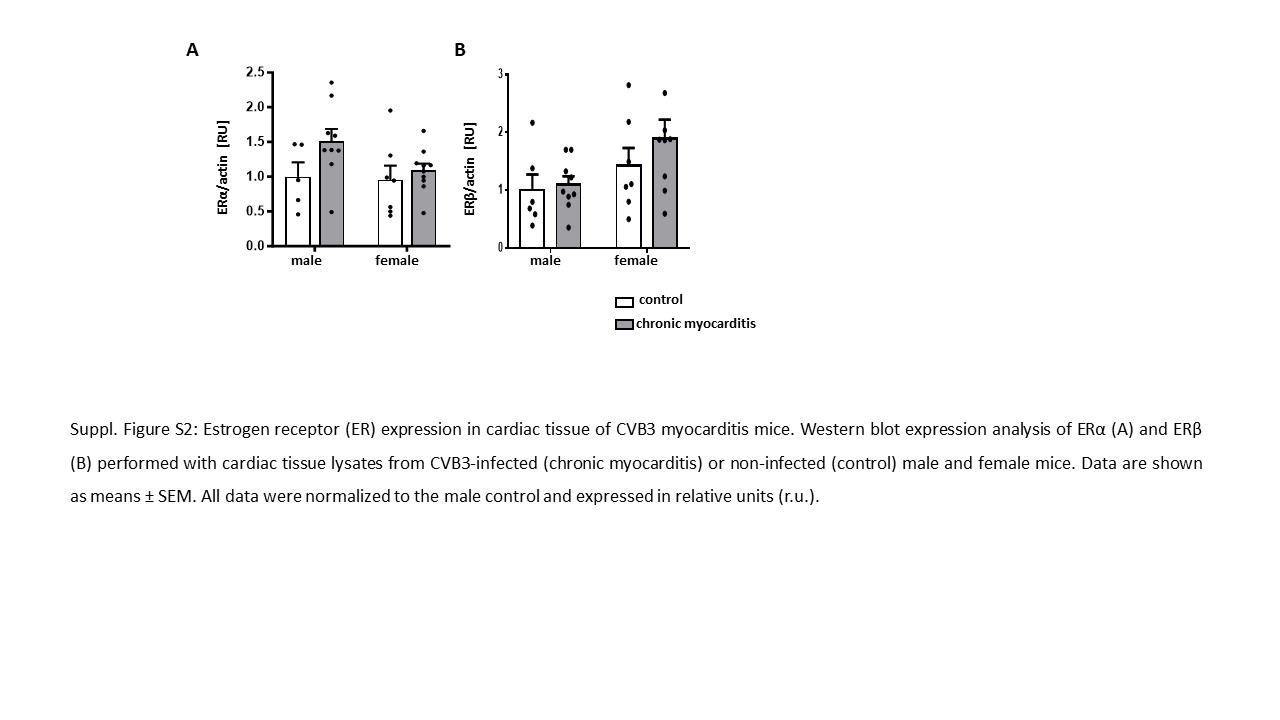

Supplement: Supplementary file 1 [file ijms-25-09666-s001.zip › Figure S2.JPG]

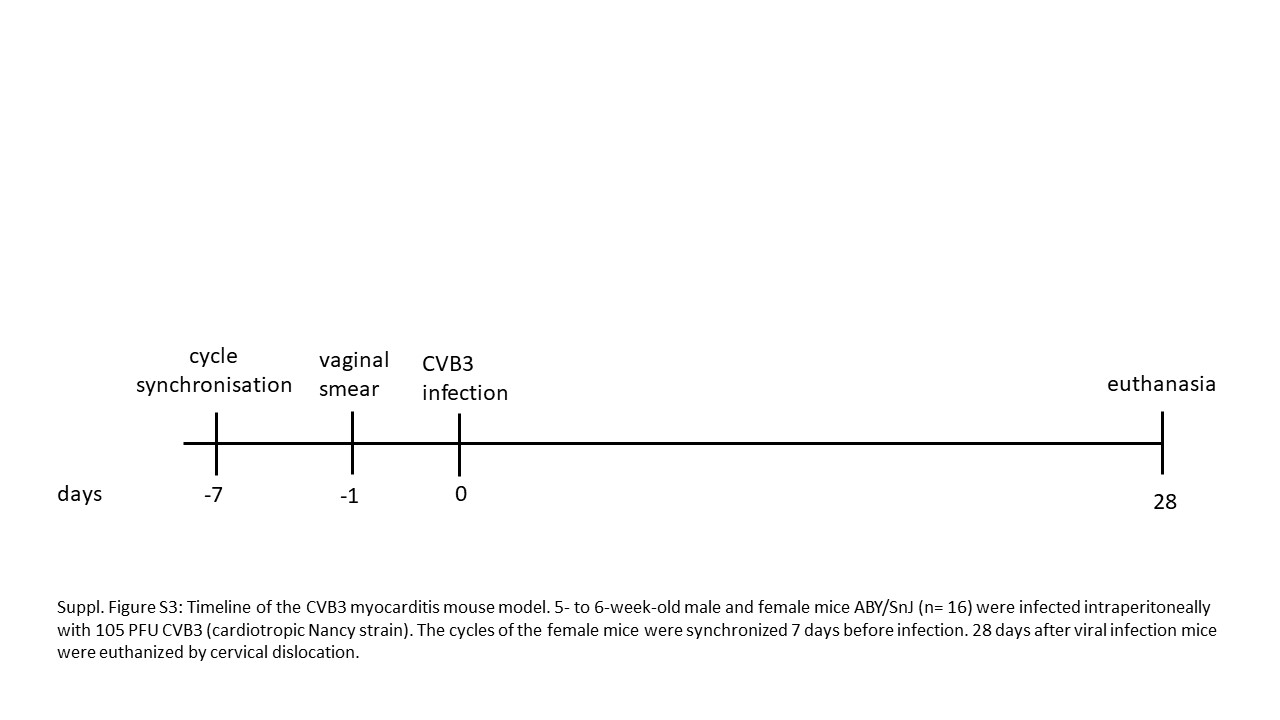

Supplement: Supplementary file 1 [file ijms-25-09666-s001.zip › Figure S3.JPG]
